# Anti-dementia drugs and changes in gait: a pre-post quasi-experimental pilot study

**DOI:** 10.1186/1471-2377-13-184

**Published:** 2013-11-21

**Authors:** Olivier Beauchet, Cyrille P Launay, Gazan Allali, Gilles Watfa, Karim Gallouj, François R Herrmann, Cédric Annweiler

**Affiliations:** 1 Department of Neuroscience, Division of Geriatric Medicine, UPRES EA 4638, UNAM, Angers University Hospital, 49933 Angers cedex 9, France; 2 Department of Neurology, Geneva University Hospital and University of Geneva, Geneva, Switzerland; 3 Department of Internal Medicine and Geriatrics, Nancy University Hospital, Nancy, France; 4 Department of Geriatrics, Tourcoing Hospital, Tourcoing, France; 5 Department of Internal Medicine, Rehabilitation and Geriatrics, Geneva University Hospitals and University of Geneva, Geneva, Switzerland; 6 Robarts Research Institute, Department of Medical Biophysics, Schulich School of Medicine and Dentistry, the University of Western Ontario, London, ON, Canada

**Keywords:** Gait, Stride time variability, Anti-dementia drugs, Alzheimer disease

## Abstract

**Background:**

Anti-dementia drugs may improve gait performance. No comparison between acetylcholinesterase inhibitors (CEIs) and memantine-related changes in gait variability has been reported. The objectives of this study were to 1) quantify and compare the mean values and coefficients of variation (CoV) of stride time in demented patients with Alzheimer’s disease and related disorders (ADRD) before and after the use of CEIs or memantine, and in age- and gender-matched controls patients with ADRD using no anti-dementia drugs; and 2) to determine whether changes in CoV of stride time differed between CEIs or memantine.

**Methods:**

A total of 120 demented patients with mild-to-moderate ADRD were prospectively included in this pre-post quasi-experimental study with two intervention groups (43 patients taking CEIs, and 41 taking memantine) and a control group (36 age- and gender matched patients without any anti-dementia drugs). CoV of stride time and walking speed were measured with GAITRite® system while usual walking at steady state. Age, gender, number of drugs daily taken, use of psychoactive drugs, body mass index and time between the two visits were also recorded.

**Results:**

There was no difference between groups for the time between baseline and follow-up assessments (232.9 ± 103.7 days for patients without anti-dementia drugs, 220.0 ± 67.5 days for patients with CEIs, 186.7 ± 96.2 days for patients with memantine, P = 0.062). Patients with memantine had a lower (i.e., better) CoV of stride time at follow-up assessment compared to those with CEIs (4.2 ± 2.4% versus 5.8 ± 4.2%, P = 0.010). Patients with memantine had a greater decrease in CoV of stride time compared to those with CEIs (−1.90% versus 0.93%, P = 0.010) and mixed-effects linear regressions showed that this decrease was specifically explained by memantine (P = 0.028).

**Conclusions:**

Our results showed that patients with ADRD and treated with memantine, but not those with CEIs, decreased their gait variability, and thus improved their gait safety (Trial registration number: NCT01315704).

## Background

Acetylcholinesterase inhibitors (CEIs) (i.e., donepezil, galantamine and rivastigimine) and NMDA receptor antagonist (i.e., memantine) are symptomatic drugs for the treatment of patients with Alzheimer’s disease and related disorders (ADRD), respectively with mild-to-moderate and moderate-to-severe stages [1-3]. The use of these drugs has proved to temporarily stabilize and/or to delay cognitive and functional declines in ADRD [1,3]. A limited number of studies have highlighted that these anti-dementia drugs may also improve gait performance [4-7]. In particular, two studies have reported a decrease in gait variability in demented patients using either donepezil or memantine [5,7]. Gait variability is defined as fluctuations in stride-to-stride intervals and may be measured by the coefficient of variation (CoV = [standard deviation/mean] × 100) of spatio-temporal gait parameters [8]. Improvements of gait variability are useful for patients since lower (i.e., better) gait variability while walking at steady state self-selected pace illustrates an efficient gait control and a safety gait [5-10]. For instance, a low stride-to-stride variability of stride time - a measure of the reliability of lower-limb movements depending on higher-levels gait control - has been associated with greater gait safety in demented patients [5-11]. To date, CEIs-related improvement of gait performance has been explained by enhancements of the attention resource allocation involved in gait control [4,5]. In parallel, memantine-related gait improvement has been explained by its dopaminergic effect [5,7,12]. However, no comparison between CEIs- and memantine-related improvements of gait variability has been performed yet in demented patients. We hypothesized that CEIs and memantine could reduce the CoV of stride time, and that this anti-dementia drug-related changes in CoV of stride time could be different between CEIs and memantine because of different mechanisms of action. Indeed, memantine has a cognitive and motor effect explained respectively by a non-competitive antagonist action on neuronal N-methyl-D-aspartate (NMDA)-type glutamate and nicotinic acetylcholine receptors combined with an agonist action on neuronal dopamine D_2_ receptors [3]. In contrast, CEIs have only a cognitive effect explained by an inhibition of acetylcholinesterase enzyme that increases both the level and duration of action of acetylcholine [1,2]. The objectives of this study were to 1) quantify and compare mean values and CoV of stride time in patients with ADRD before and after the use of CEIs or memantine, and in age- and gender-matched controls with ADRD using no anti-dementia drugs; and 2) to determine whether changes in CoV of stride time differed between CEIs or memantine.

## Methods

### Participants and assessment

Between June 2011 and December 2012, 84 demented patients with mild-to-moderate ADRD (mean age 82.2 ± 6.5 years; 65.5% female) with CEIs (n = 43) and memantine (n = 41), and 36 age- and gender-matched controlled demented patients with mild-to-moderate ADRD without treatment (mean age 81.3 ± 5.5 years; 61.1% female) were prospectively and consecutively included in this quasi-experimental study (Trial registration number: NCT01315704). The assignment in both intervention groups (i.e., participants with CEIs and participants with memantine) was not randomized and it was an open label study. The choice of the anti-dementia drug was based on the severity of the cognitive decline (mild-to-moderate for CEIs, and moderate for memantine), contraindications and side effects of CEIs and memantine. The age (plus or minus 2 years) and gender matching were performed only on the control group (i.e., participants without anti-dementia drugs). Inclusion criteria were outpatients visiting the memory clinic with a de novo diagnosis of mild-to-moderate ADRD and at least one follow-up visit with two gait analyses separated by at least 6 months in the memory clinic of Angers University Hospital, France. At baseline assessment, all participants had no anti-dementia drugs. In addition, those receiving an anti-dementia drug during the follow-up (i.e., the intervention group) had an effective daily dose (i.e., at least 5 mg of donepezil, 16 mg galantamine, 9.6 mg rivastigmine patch, and 20 mg memantine) for at least 3 months. Participants with co-prescription of cerebral vasodilatators, renal failure, extrapyramidal rigidity of the upper limbs (score above 2 on item 22 of the Unified Parkinson’s Disease Rating Scale motor score) [13], acute medical illness within the past month, severe orthopaedic diagnoses, depression (i.e., 4-item Geriatric Depression Scale ≥1) [14], as well as those using walking aids and anticholinergic medication were excluded. Four hundred and twelve patients were followed during the period of inclusion and 219 (53.2%) met the selection criteria. Among this subgroup, 143 (65.3%) took an anti-dementia drug but only 84 (38.4%) at an effective dose. Among the 76 (34.7%) participants who did not take anti-dementia drugs, 36 (16.4%) were included based on the matching criteria. Having a group of patients without anti-dementia drugs was possible due to the 6-month period corresponding to the delay of paraclinical investigations required for the prescription of AD-specific treatment in the memory clinic of Angers University Hospital. For each patient included in the study and who had an anti-dementia drug, one matched patient with no anti-dementia drugs was included. All included participants after this process of selection completed the study.

Participants included in the study underwent neurological examination, neuropsychological testing, and brain imaging. In addition, age, gender, number of drugs daily taken, use of psychoactive drugs (i.e., benzodiazepines, antidepressants or neuroleptics), height (cm), weight (kg) and time (day) between the two visits were recorded. The body mass index (kg/m^2^) was calculated. The diagnoses of ADRD were made during multidisciplinary meetings involving geriatricians, neurologists and neuropsychologists. The diagnosis of ADRD followed the Diagnostic and Statistical Manual of Mental Disorders, 4^th^ edition and the National Institute of Neurological and Communicative Disorders and Stroke and the Alzheimer's Disease and Related Disorders Association criteria [15]. The CoV of stride time, calculated with the following formula CoV=[Standard deviation/mean] × 100 (the stride time being the time elapsed between the contact of two consecutive footsteps of the same foot), and walking speed were measured using GAITRite® system (GAITRite Gold walkway, 972 cm long, active electronic surface area 792×610 cm, with a total of 29,952 pressure sensors, scanning frequency 60 Hz, software version 3.8, CIR System, Havertown, PA). The participants were asked to walk straight ahead at their usual self-selected walking speed. Each participant completed one trial. Participants walked in a quiet, well-lit room wearing their own footwear according to European guidelines for spatio-temporal gait analysis in older adults [16].

### Standard protocol approvals, registrations, and patient consents

The study was conducted in accordance with the ethical standards set forth in the Helsinki Declaration (1983). The entire study protocol was approved by the local Ethical Committee of Angers (France). Written informed consent for participation in the study was obtained from all participants.

### Statistics

The participants’ baseline characteristics were summarized using means and standard deviations or frequencies and percentages, as appropriate. The normality of the parameters’ distribution was verified with a Shapiro-Francia test before and after applying usual transformations to normalize non-Gaussian variables. Participants were separated into 3 groups: those without anti-dementia drugs, those using CEIs, and those using memantine. First, between-group comparisons were performed using analysis of variance the Kruskal-Wallis, Mann–Whitney or Chi-square tests, as appropriate. Second, anti-dementia drugs effect (i.e., no drugs versus drugs), time effect (i.e., time between before and after anti-dementia drugs use) and an anti-dementia drugs effect X time effect interaction were included as independent variables in a repeated analysis of covariance (ANCOVA) to analyze their respective influence on CoV of stride time (dependent variable), with and without adjustment on baseline characteristics of participants. Third, a mixed-effect linear regression model (with Stata "xtmixed" command) using the same variables was performed to specify which anti-dementia drugs explained the change in CoV of stride time. P-values <0.05 were considered statistically significant. All statistics were performed using SPSS (version 15.0; IBM, Inc., Chicago, IL) and Stata (version 12.1; College Station, TX).

## Results

There was no difference between groups for the time between baseline and follow-up assessments (P = 0.062) (Table 1). Between-group comparisons showed that there was a significant difference for MMSE score (P < 0.001) and CoV of stride time after treatment (P = 0.035). Demented patients using memantine had a lower MMSE score at baseline compared to those with CEIs (P < 0.001) and to those without anti-dementia drug (P < 0.001). Patients treated with memantine had a lower CoV of stride time at follow-up assessment compared to those with CEIs (P = 0.010). There was no significant difference between groups for the other baseline characteristics.

**Table 1:** Characteristics and comparisons of the participants’ characteristics separated into three groups according to the type of anti-dementia drug used (n = 120)

	**No treatment (n = 36)**	**CEIs (n = 43)**	**Memantine (n = 41)**	P-value^*^			
				**Overall**	**No treatment versus CEIs**	**No treatment versus memantine**	**CEIs versus memantine**
Age, mean ± SD (years)	81.3 ± 5.5	81.0 ± 6.6	83.4 ± 6.3	0.189	-	-	-
Female gender, n (%)	22 (61.1)	30 (69.8)	25 (61.0)	0.633	-	-	-
BMI, mean ± SD (kg/m^2^)	26.9 ± 4.4	26.2 ± 4.9	26.1 ± 4.5	0.763	-	-	-
Number of drugs daily taken, mean ± SD	6.2 ± 3.2	5.2 ± 3.3	6.0 ± 3.1	0.267	-	-	-
Use psychoactive drugs^†^, n (%)	17 (47.2)	16 (37.2)	12 (29.3)	0.348			
MMSE score‡ (/30 points), mean ± SD	20.8 ± 5.7	19.8 ± 4.6	14.7 ± 4.3	**<0.001**	0.169	**<0.001**	**<0.001**
Time between visits, mean ± SD (days)	232.9 ± 103.7	220.0 ± 67.5	186.7 ± 96.2	0.062	-	-	-
Walking speed (cm/s), mean ± SD	68.3 ± 21.3	62.4 ± 21.3	60.9 ± 22.8	0.466	-	-	-
CoV of stride time (%), mean ± SD							
Before treatment	4.8 ± 2.2	4.9 ± 2.8	6.1 ± 5.0	0.699	-	-	-
After treatment	5.4 ± 5.7	5.8 ± 4.2	4.2 ± 2.4	**0.035**	0.084	0.647	**0.010**

CEIs: acetylcholinesterase inhibitors; BMI: body mass index; MMSE: Folstein's Mini-Mental State Examination; CoV: coefficient of variation; SD: standard deviation; *: comparison based on analysis of variance Kruskal-Wallis test, Mann–Whitney or the Chi-square test, as appropriate; †: benzodiazepines, antidepressants or neuroleptics at baseline assessment; ‡: at baseline assessment (i.e., before treatment); P-value significant (i.e., <0.05) indicated in bold.

**Figure 1:**
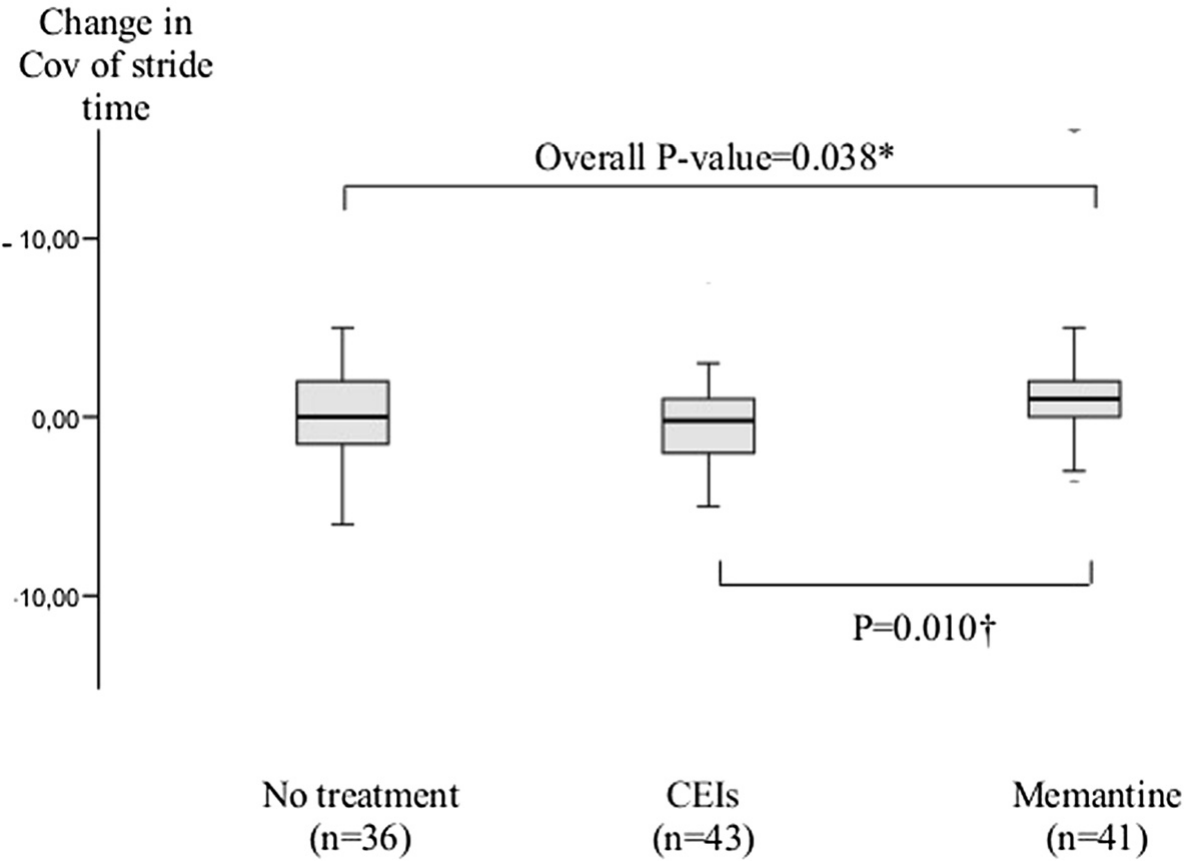
**Mean value and standard deviations of change in CoV of stride time between before and after treatment according to treatment groups (n=120).** CEIs: acetylcholinesterase inhibitors: CoV: coefficient of variation; *: Comparison based on Kruskal-Wallis test; †: Comparison based on Mann-Whitney test.

Between-group comparison of the change in CoV of stride time between baseline and at follow-up assessment was significant (P = 0.038) but only patients with memantine had a higher change compared to those with CEIs (P = 0.010) (Figure 1). There was no significant difference between participants without anti-dementia drugs and those using CEIs (P = 0.288) and those using memantine (P = 0.176).

As shown in Table 2, the ANCOVA showed that anti-dementia drugs decreased CoV of stride time only while taking in consideration the time effect (P = 0.034 for model non-adjusted on baseline characteristics, and P = 0.038 for full adjusted model). In final, mixed-effects linear regressions underlined that anti-dementia drug-related decrease in CoV of stride time was explained by memantine (P = 0.028) but not CEIs (P > 0.250) (Table 3). Moreover, full adjusted model showed that female gender (P = 0.046) and a high MMSE score at baseline assessment (P = 0.003) were associated with a significant decrease of CoV of stride time.

**Table 2:** Results of analysis of covariance with a repeated measures (n = 240) design analyzing the influence of anti-dementia drugs effect (i.e., no anti-dementia drug, acetylcholinesterase inhibitors or memantine), time effect (i.e., time between before and after anti-dementia drugs introduction) and their interaction on the coefficient Cov of stride time (dependent variable) among participants (n = 120)

**Source of variation**	CoV of stride time*					
	**Model 1†**			**Model 2†**		
	**P-value‡**	**df**	**F**	**P-value‡**	**df**	**F**
Anti-dementia drugs effect#	0.937	2.5	0.06	0.612	2.5	0.50
Time effect¶	0.608	1.5	0.26	0.606	1.5	0.27
Anti-dementia drugs x time interaction	**0.034**	2.5	3.43	**0.038**	2.5	3.50
Age				0.850	1.5	0.04
Female gender				0.958	1.5	0.00
BMI				0.078	1.5	3.23
Number of drugs daily taken				0.857	1.5	0.03
Use of psychoactive drugs**				0.918	1.5	0.01
MMSE score††				0.093	1.5	2.94
Walking speed				0.265	1.5	1.27

CoV: coefficient of variation; BMI: body mass index; df: degree of freedom; MMSE: Folstein's Mini-Mental State Examination; *: normalized by taking the square-root and coded as a continuous variable, †: separated models (Model 1: non-adjusted on baseline characteristics; Model 2: full adjusted model); ‡: box conservative estimate; #: pool effect of acetylcholinesterase inhibitors and memantine; ¶: time between before and after introduction of anti-dementia drugs; **: benzodiazepines, antidepressants or neuroleptics; ††: at baseline assessment (i.e., before treatment); P-value significant (i.e., P < 0.05) indicated in bold.

**Table 3:** Mixed-effects linear regressions predicting the change in CoV of stride time (n = 240 measures and n = 120 participants)

	Change in CoV of stride time*					
	**Model 1†**			**Model 2†**		
	**β**	**95% CI**	**P-value**	**β**	**95% CI**	**P-value**
Drugs effect						
Use of CEIs	−0.067	[−0.381; 0.246]	0.674	−0.193	[−0.512; 0.125]	0.234
Use of memantine	0.179	[−0.138; 0.496]	0.268	−0.014	[−0.341; 0.313]	0.933
Time effect‡	0.019	[−0.232; 0.269]	0.885	0.019	[−0.222; 0.259]	0.878
Drugs x time interaction						
No treatment	Ref			Ref		
Use of CEIs	0.186	[−0.153; 0.526]	0.282	0.190	[−0.138; 0.518]	0.256
Use of memantine	−0.385	[−0.728; -0.415]	**0.028**	−0.371	[−0.702; -0.041]	**0.028**
Age				0.000	[−0.000; 0.000]	0.811
Female gender				−0.229	[−0.453; -0.004]	**0.046**
BMI				−0.0004	[−0.0000; 0.0008]	0.053
Number of drugs daily taken¶				0.011	[−0.019; 0.041]	0.490
Use of psychoactive drugs#¶				0.081	[−0.278; 0.116]	0.422
MMSE score¶				−0.028	[−0.046; -0.009]	**0.003**
Walking speed¶				−0.003	[−0.006; 0.0001]	0.147

CI = confidence interval; CEIs: acetylcholinesterase inhibitors; BMI: body mass index; MMSE: Folstein's Mini-Mental State Examination; CoV: coefficient of variation; β: coefficient of regression beta corresponding to change in CoV of stride time; *: normalized by taking the square-root and coded as a continuous variable; †: separated models (Model 1: non-adjusted on baseline characteristics; Model 2: full adjusted model); ‡: time between before and after introduction of anti-dementia drugs; #: benzodiazepines, antidepressants or neuroleptics at baseline assessment; ¶: at baseline assessment (i.e., before treatment); P-value significant (i.e., P < 0.05) indicated in bold.

## Discussion

Our results showed that memantine, but not CEIs, decreases gait variability in patients with ADRD. This memantine-related improvement of gait variability was shown few months after the first introduction of drug and confirms a recent study reporting similar results but without a comparison group with CEIs [7]. It may be related to specific effects of memantine on both subcortical and cortical levels of gait control. Indeed, the improvement of gait variability with memantine may be explained by its dopamergic effects, which improve extrapyramidal motricity by acting on the dopamine D2 receptors [3,7]; but also by its glutamatergic effects on the cognitive field, specifically the higher levels of gait control [5,6,10]. Regarding to the progression of the extrapyramidal syndrome during the course of ADRD [17] and the fact that the patients treated with memantine presented a lower MMSE, gait improvement presented in this group could be related with a specific effect of memantine on the extrapyramidal system.

No significant gait improvement with CEIs was shown in our study. Opposite results were already published about gait improvement due to CEIs. For instance, a CEIs-related decrease in gait variability has been reported by Montero-Odasso et al. while single- and dual-tasking [5]. However it is of note that, similarly to our results, there were no significant changes in gait performance while usual walking in patients treated with galantamine in Assal et al.’s study [6]. In the latter study, the authors still retained a gait improvement because non-treated controls suffered a significant dual-task decrement in stride time compared to cases using galantamine. These previous results suggested that CEIs could improve gait performance mainly while dual-tasking rather than single-tasking. The principle of the dual-task paradigm is to examine gait performance while simultaneously executing an attention-demanding task [18]. Performance changes in dual-task compared to single-task are usually interpreted as interference due to competing demands for attention resources needed for both tasks and mainly depend on one’s ability to properly allocate attention between the two tasks [9,11,18]. Previous results thus strengthen the idea that CEIs may improve the cognitive component of gait, with gait improvements especially identifiable in dual-task. As a consequence, further research examining gait performance while single- and dual-tasking is needed to better understand the exact effects of memantine and CEIs on gait. In final, our results showed that memantine-related decrease in gait variability was associated with the level of global cognitive functioning, a higher level being associated with a greater decrease. This result may be explained by the cognitive and motor effects of memantine, and underscores that, when ADRD is at a severe stage, symptomatic effects of memantine are limited, probably because of the diffuse neurodegenerative lesions in the brain.

Some limitations of this study need to be considered. Firstly, the limited number of participants from one single memory clinic may be unrepresentative of the general population of patients with ADRD. Second, the pre-test/post-test quasi-experimental open-label design with no randomization of the assignment of participants into intervention and control groups, and without a placebo group, may limit the interpretation of our results. Third, although we were able to control for the main characteristics likely to modify the association between the change of MMSE score and the double treatment arm, residual confounders might still be present. Finally, additional limitations lies in the failure to consider other dementias such as vascular dementia and regarding the improvement in the memantine group a regression to the mean phenomenon can never be completely excluded even if it seems unlikely.

## Conclusion

In conclusion, we found a memantine-related decrease in gait variability, and thus an improvement of gait safety, among patients with ADRD. An ongoing double-blind randomized placebo-controlled parallel group intent-to-treat superiority clinical trial, the AD-IDEA trial (ClinicalTrials.gov number: NCT01409694) [19], is conducted to investigate whether the memantine-related decrease in gait variability is confirmed.

## Abbreviations

CEIs: Acetylcholinesterase inhibitors; ADRD: Alzheimer’s disease and related disorders; ANCOVA: Analysis of covariance; CoV: Coefficient of variation; MMSE: Mini mental status examination; NMDA: N-methyl-D-aspartate.

## Competing interests

The study was financially supported by Lundbeck France pharmaceutical company. The sponsors had no role in the design and conduct of the study, in the collection, management, analysis, and interpretation of the data, or in the preparation, review, or approval of the manuscript. All authors declare that they have no competing interests.

## Authors’ contributions

OB, CL and CA participated in designing the study, writing and reviewing of the manuscript. GA, FRH, GW, KG participated in reviewing the manuscript. All authors read and approved the final manuscript.

## Pre-publication history

The pre-publication history for this paper can be accessed here:

http://www.biomedcentral.com/1471-2377/13/184/prepub

## References

[B1] Tsuno N Donepezil in the treatment of patients with Alzheimer's disease Expert Rev Neurother 2009 13 591 598 10.1586/ern.09.23 19402770

[B2] Onor ML Trevisiol M Aguglia E Rivastigmine in the treatment of Alzheimer's disease: an update Clin Interv Aging 2007 13 17 32 10.2147/ciia.2007.2.1.17 18044073 PMC2684084

[B3] McKeage K Memantine: a review of its use in moderate to severe Alzheimer's disease CNS Drugs 2009 13 881 897 10.2165/11201020-000000000-00000 19739697

[B4] Montero-Odasso M Wells JL Borrie MJ Speechley M Can cognitive enhancers reduce the risk of falls in older people with mild cognitive impairment? A protocol for a randomised controlled double blind trial BMC Neurol 2009 13 42 10.1186/1471-2377-9-42 19674471 PMC3224736

[B5] Montero-Odasso M Wells J Borrie M Can cognitive enhancers reduce the risk of falls in people with dementia? An open-label study with controls J Am Geriatr Soc 2009 13 359 360 10.1111/j.1532-5415.2009.02085.x 19207156 PMC5017867

[B6] Assal F Allali G Kressig RW Herrmann FR Beauchet O Galantamine improves gait performance in patients with Alzheimer's disease J Am Geriatr Soc 2008 13 946 947 10.1111/j.1532-5415.2008.01657.x 18454755

[B7] Beauchet O Allali G Launay C Fantino B Annweiler C Does memantine improve the gait of individuals with Alzheimer's disease? J Am Geriatr Soc 2011 13 2181 2182 10.1111/j.1532-5415.2011.03648.x 22098042

[B8] Annweiler C Beauchet O Bartha R Wells JL Borrie MJ Hachinski V Montero-Odasso M Motor cortex and gait in mild cognitive impairment: a magnetic resonance spectroscopy and volumetric imaging study Brain 2013 13 859 871 10.1093/brain/aws373 23436505

[B9] Beauchet O Annweiler C Montero-Odasso M Fantino B Herrmann FR Allali G Gait control: a specific subdomain of executive function? J Neuroeng Rehabil 2012 13 12 10.1186/1743-0003-9-12 22321772 PMC3308913

[B10] Rosano C Brach J Longstreth WT Jr Newman AB Quantitative measures of gait characteristics indicate prevalence of underlying subclinical structural brain abnormalities in high-functioning older adults Neuroepidemiology 2006 13 52 60 10.1159/000089240 16254454

[B11] Beauchet O Allali G Annweiler C Bridenbaugh S Assal F Kressig RW Herrmann FR Gait variability among healthy adults: low and high stride-to-stride variability are both a reflection of gait stability Gerontology 2009 13 702 706 10.1159/000235905 19713694

[B12] Almeida QJ Frank JS Roy EA Patla AE Jog MS Dopaminergic modulation of timing control and variability in the gait of Parkinson's disease Mov Disord 2007 13 1735 1742 10.1002/mds.21603 17557356

[B13] Ebersbach G Baas H Csoti I Müngersdorf M Deuschl G Scales in Parkinson's disease J Neurol 2006 13 iv32 iv35 10.1007/s00415-006-1108-9 16944355

[B14] Shah A Herbert R Lewis S Mahendran R Platt J Bhattacharyya B Screening for depression among acutely ill geriatric inpatients with a short geriatric depression scale Age Ageing 1997 13 217 221 10.1093/ageing/26.3.217 9223718

[B15] McKhann G Drachman D Folstein M Katzman R Price D Stadlan EM Clinical diagnosis of Alzheimer's disease: report of the NINCDS-ADRDA work group under the auspices of department of health and human services task force on Alzheimer's disease Neurology 1984 13 939 944 10.1212/WNL.34.7.939 6610841

[B16] Kressig RW Beauchet O Guidelines for clinical applications of spatio-temporal gait analysis in older adults Aging Clin Exp Res 2006 13 174 176 10.1007/BF03327437 16702791

[B17] Stavitsky K Brickman AM Scarmeas N Torgan RL Tang MX Albert M Brandt J Blacker D Stern Y The progression of cognition, psychiatric symptoms, and functional abilities in dementia with Lewy bodies and Alzheimer disease Arch Neurol 2006 13 1450 1456 10.1001/archneur.63.10.1450 17030662

[B18] Beauchet O Allali G Berrut G Hommet C Dubost V Assal F Gait analysis in demented subjects: Interests and perspectives Neuropsychiatr Dis Treat 2008 13 155 160 18728766 10.2147/ndt.s2070 PMC2515920

[B19] Annweiler C Fantino B Parot-Schinkel E Thiery S Gautier J Beauchet O Alzheimer's disease–input of vitamin D with mEmantine assay (AD-IDEA trial): study protocol for a randomized controlled trial Trials 2011 13 230 10.1186/1745-6215-12-230 22014101 PMC3212921

